# Breaking down barriers: promoting journals beyond the page with open access journal clubs

**DOI:** 10.1192/bjb.2024.3

**Published:** 2025-02

**Authors:** Angharad N. de Cates, Donncha Mullin, Lucy Stirland, Mariana Pinto da Costa, Derek Tracy

**Affiliations:** 1 University of Oxford, Oxford, UK; 2 Oxford Health NHS Foundation Trust, Oxford, UK; 3 University of Edinburgh, Edinburgh, UK; 4 Institute of Psychiatry, Psychology and Neuroscience, King's College London, London, UK; 5 West London NHS Trust, London, UK

**Keywords:** Education and training, medical technology, patient/carer involvement, statistical methodology, ethics

## Abstract

In 2020, during the early days of the COVID-19 pandemic, the *British Journal of Psychiatry* (*BJPsych*) established a series of free online teaching sessions called *BJPsych* Journal Clubs. Their educational purpose is two-fold: (a) to provide junior psychiatrists with a friendly but large-scale platform to evaluate and critically appraise recent articles published in the *BJPsych* and (b) to present new research findings in an open and accessible manner. In this paper, we discuss our framework, the challenges we encountered, how the original model is evolving based on feedback from trainees, and tips for success when delivering international online journal clubs.

The Royal College of Psychiatrists' (RCPsych) *British Journal of Psychiatry* (*BJPsych*) is a high-impact scientific journal. Aligned to the RCPsych's charitable status and aims, it has a broader aim to engage and educate a diverse audience on contemporary issues and advances in mental health and neuroscience. As part of this, in 2020 *BJPsych* editorial board members Derek Tracy and Angharad de Cates established the *BJPsych* Journal Clubs. As well as further disseminating discussed research papers from the *BJPsych*, these online events aimed to support junior psychiatrists and early-career researchers in presenting a research paper as part of local clinical teaching activities. This is somewhat akin to ‘traditional’ journal clubs typically held face-to-face at local trainee teaching, but with enhancements that online formats and the support of a leading scientific journal can provide,^1,2^ as all journal clubs are dependent on the skills and experience of the supervisory team. The *BJPsych* team also designed these journal clubs to amplify the work to a broader audience, including through involving authors of the discussed article and other international experts on the topic, and linking with the RCPsych and other institutions. In particular, it was intended that the audience should include healthcare students and trainee psychiatrists and that the journal clubs should help grow the trainee presenters’ research experience and confidence in undertaking a national/international presentation. Finally, the educational legacy of the journal club was important: to ensure that clinicians and researchers in the audience had time to consider how this paper might move a clinical/research field forwards and what relevance this might have to a clinical scenario today.

## The format of a *BJPsych* Journal Club

The sessions are co-hosted by both a *BJPsych* representative and a local organiser, who together plan, chair, introduce and explain the programme to the audience. Particular attention is paid to ensuring diversity within the overall panel, including, but not limited to, gender and ethnicity. After introductions, trainee speakers give a 20 min presentation of the paper and a brief critical appraisal. An expert panel of three senior academics follow with their reaction and reflections on the paper. Usually an author from the paper is included either live or by pre-recorded message (Box 1 gives reflections of an author who attended live). The discussion is then opened to the online audience for typed ‘chat’ or verbal questions and comments, with the chairs moderating questions before bringing the event to a close with a recap of the ‘take home’ messages of the session.

Box 1 A reflection from an author involved in a *BJPsych* Journal Club

‘My experience of participating in the *BJPsych* was overwhelmingly positive.

The journal club's format, where accomplished PhD candidates presented my work for analysis by practitioners with clinical, methodological, and statistical expertise, initially appeared to be a formidable endeavour for an author. However, as the discussions unfolded, it became apparent that the organizers had cultivated an environment that supported constructive dialogue.

I was impressed by the depth of knowledge and insight exhibited by the presenters as they engaged with and dissected my work. Equally captivating was the discourse between experts from diverse fields. The journal club experience not only afforded me invaluable insights into how my work is perceived and understood by others, it also yielded specific and actionable feedback regarding the methodological approaches I currently employ.

Overall, the journal club contributed not only to my understanding of how my work resonates but also to enhancing the precision of my methodological practices.’

Good team-working and appropriate delegation of tasks is essential when organising an online conference or academic event.^3^ To prepare for each journal club, the *BJPsych* Journal Club team works with a partner organisation to form a session-specific team for co-production of the session. Initially, partner organisations were focused in the UK, with a later expansion internationally. The partner organisation is responsible for finding presenters and suggesting panel members. The *BJPsych* provides support with the online platform, advertising and technical expertise on the paper as well as supporting choosing an expert panel.

Selected presenters then join the session-specific Journal Club team to choose an appropriate article for presentation. The article must have been published in the *BJPsych* but can feature any topic or methodology and can be recent or historic. Ensuring presenters are involved in this choice helps to reduce any anxiety they might feel when presenting to a potentially large audience in an online forum.

## The outline of journal club sessions undertaken so far

So far, we have run six journal clubs, intentionally spread around the UK, Ireland and beyond: Birmingham in 2020; London (the UCL Mental Health MSc group) and Dublin in 2021; Edinburgh and Cardiff in 2022; and most recently with the World Psychiatric Association Early Career Psychiatrists section in 2023.

For the first pilot session, audience members required a university or National Health Service (NHS) email address to register, to ensure that any demand for tickets could be met and to facilitate moderation. But subsequently, successful running of initial sessions enabled us to widen audience participation to anyone available to receive an Eventbrite link. This includes international colleagues and members of the public as participants, following social media requests for greater inclusivity.

As social media are a low-intensity resource for global distribution of information, advertising for the journal clubs has occurred primarily using Twitter/X with tweets sent from the *BJPsych* account at increasing frequency in the fortnight prior to the session and retweeted by the organising team to maximise distribution. The global nature of social media advertising allows the *BJPsych* Journal Clubs to have the broadest reach possible, while being aware of possible challenges in communicating through these media, such as word limits and potential for misunderstandings.^4,5^

Between 2020 and 2023, we also progressed from a standard online meeting to a webinar as that platform appeared to provide the correct balance of ease of use versus security.

## General session challenges

There are some challenges related to recording of the sessions. First, the very act of recording risks altering the dynamic of the sessions, through increasing stress for junior participants, potentially reducing freedom of expression or at least making participants and audience members more careful and stilted in their approach, and, conversely, opening the question of responsibility should anything offensive or litigious be said. We therefore aimed to make the environment as open and informal as possible within these limitations, including an avoidance of titles when referring to panellists.^1^ We also ensured that all appraisers had a rehearsal within the electronic hosting platform in front of the organising team to practise their presentation, anticipate timing and allow familiarisation. Second, there was a practical question of a suitable location for recordings to be archived and stored to ensure easy availability. To enable all resources from each session to be easily discovered after the live session, we have created a specific and searchable location from the main *BJPsych* website: the Magnify blog site (see below).

Another challenge was keeping the content of the session to an hour. After four sessions, it became apparent that the panel questions and discussion are an important strength of the journal clubs. To ensure sufficient time, we have found it is necessary for the chair to keep the appraisal component to a strict maximum of 20 min, which is sufficient time to relay the key points of the paper for those not familiar with it beforehand but also prevents restriction of audience participation.

## The post-journal club survey

**Fig. 1:**
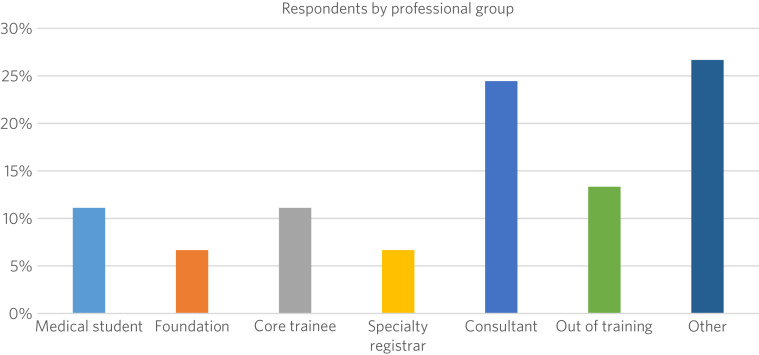
Respondents to the *BJPsych* Journal Club surveys across the four sites. ‘Other’ refers to *n* = 2 respondents from each of the following categories: PhD student, MSc student, doctoral student, psychology student and researcher.

A total of 45 respondents from the first four events (Birmingham, London, Dublin, Edinburgh) completed a post-journal club survey (the survey questions are shown in the Supplementary material, available online at https://dx.doi.org/10.1192/bjb.2024.3). Approximately one-quarter of respondents were consultants, and the remainder were healthcare students (including PhD, MSc and undergraduate psychology students), trainees and specialist and associate specialist (SAS) doctors (Fig. 1).

### Ethics statement

The survey did not require ethical approval as the data collected were an optional evaluation of teaching. Written informed consent to use anonymised information from the survey when evaluating or writing reports regarding *BJPsych* Journal Clubs was obtained from all respondents. Taking part in the survey was optional for attendees of the journal clubs.

### Summary of feedback

Three-quarters of all respondents rated the speakers as highly engaging, scoring the speakers as ≥75% out of 100%. The organisation and structure of the sessions were also generally highly regarded. The majority (82%) of respondents rated the sessions as ‘very good’ for organisation and structure.

When asked what they most liked about the event, responses centred on having the author(s) of the evaluated paper present, having expert panellists and having a wide audience. Example responses include:

‘The paper was relevant, difficult statistics were explained clearly, and the contributions of discussants and audience were well handled’.

‘Great mix of students, panellists, authors and audience. An exemplar of how to run a journal club’.

‘Having the author present who addressed the questions/ points raised’.

**Fig. 2:**
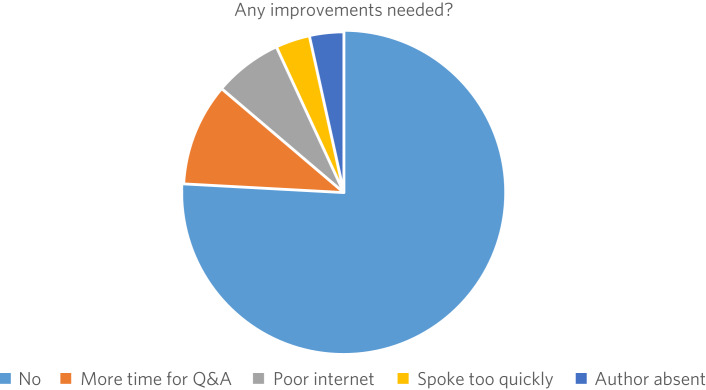
Suggested improvements to the *BJPsych* Journal Clubs. Q&A, questions and answers.

Figure 2 illustrates responses to the question ‘Was there anything about the event you disliked or felt could be improved upon?’ Over three-quarters of respondents answered ‘No’. The main improvement suggested was for more time for questions and answers in the session.

The overwhelming majority of respondents were interested in attending another *BJPsych* Journal Club (98%).

## The future of the *BJPsych* Journal Clubs

We have four ways in which we anticipate developing the next iteration of the *BJPsych* Journal Clubs, including incorporating the feedback received.

An early adjustment was to invite an author to be present at the session, directly responding to participant feedback. Authors have universally accepted invitations to attend, and the presenters and audience have always appreciated hearing their motivations for the paper and their criticisms of their own work. We have also encouraged hosts and presenters to adhere strictly to time restraints to allow the maximum opportunity for the panel to answer questions, as this always provokes interesting discussions.

We recently organised our first ‘international’ journal club co-hosted with the World Psychiatric Association (WPA). This has allowed us to develop our representation in an international direction and to further broaden our audience – making the most of the opportunities afforded by the online capabilities.^1^ We are now planning a journal club with a group of displaced Ukrainian medical students in association with the Crisis Rescue Foundation, allowing us to contribute directly to the charitable aims of the *BJPsych*.

We expect to continue running the journal clubs online to maximise the inclusivity and availability of events, as well as their greater ease and lower cost of organisation. We are mindful that there is a certain amount of interaction that is lost in an online-only event^3^ and so we plan to introduce hybrid events with a face-to-face panel in the future.

A very important development aim is to include patients and/or carers in all sessions. The most recent iteration of the journal club co-hosted with the WPA included the founder and chief executive officer of the Global Mental Health Peer Network. This allowed us to contextualise the real-world representation of mental illness, its treatments and research priorities, and continuing this will enable us to broaden the diversity of the *BJPsych* reach.

Finally, as mentioned above, we have just launched a centralised location to pool resources from the *BJPsych* Journal Clubs – the Magnify blog site (www.cambridge.org/core/blog/tag/magnify-the-journal-club-from-BJPsych). This ensures a record of all past and future *BJPsych* Journal Clubs and that the hard work of session-specific teams, presenters and panel members can be harnessed by those (often including individuals from under-represented groups) unable to attend the session itself.

## Supporting information

de Cates et al. supplementary material de Cates et al. supplementary material

## Data Availability

The data that support the findings of this study are available on request from the corresponding author A.N.d.C. The data are not publicly available as we did not obtain permission for participant data to be openly shared.

## About the authors

**Angharad N. de Cates** is an NIHR clinical lecturer in psychiatry at the University of Birmingham, UK and at the time of this article was a Guarantors of Brain clinical postdoctoral fellow in the Department of Psychiatry, Warneford Hospital, University of Oxford, UK and a specialty registrar in general adult psychiatry with Oxford Health NHS Foundation Trust, Oxford, UK. **Donncha Mullin** is a specialist registrar in the University of Edinburgh's Centre for Clinical Brain Sciences, Royal Edinburgh Hospital, Edinburgh, UK. **Lucy Stirland** is a consultant psychiatrist in the University of Edinburgh's Centre for Clinical Brain Sciences, Royal Edinburgh Hospital, Edinburgh, UK. **Mariana Pinto da Costa** is a consultant psychiatrist and a senior lecturer at the Institute of Psychiatry, Psychology and Neuroscience, King's College London, UK. **Derek Tracy** is a consultant psychiatrist and Clinical Director with West London NHS Trust, London, UK and a senior lecturer in the Department of Psychosis Studies at the Institute of Psychiatry, Psychology and Neuroscience, King's College London, UK.
