# Supplementary material for: Breaking down barriers: promoting journals beyond the page with open access journal clubs
Source: BJPsych Bull. 2025 Feb;49(1):57–60. doi: 10.1192/bjb.2024.3 (PMC11810480; doi:10.1192/bjb.2024.3)
Supplement: de Cates et al. supplementary material [file S2056469424000032sup001.pdf]

Breaking Down Barriers: Promoting journals beyond the page with open access Journal Clubs

Contents Page

1. Questionnaire items

Questionnaire items

1) What is your current stage of training?
   Medical student / Foundation trainee / Core trainee / Specialty Registrar / Consultant / Out of training / Other
2) How useful did you feel this event was in complementing your training and professional development (out of 100%)?
   <25% / 25-49% / 50 – 74% / >75%
3) How engaging do you feel the speakers were (out of 100%)?
   <25% / 25-49% / 50 – 74% / >75%
4) How organised and clearly structured did you feel the event was (out of 100%)?
   <25% / 25-49% / 50 – 74% / >75%
5) How appropriate and well selected did you feel the paper was? Were there any aspects of it which could have been explained better? *(free text)*
6) What did you like most about the event? *(free text)*
7) Was there anything about the event you disliked or felt could be improved upon? *(free text)*
8) Was there any more information that would have been helpful to have before the event? *(free text)*
9) Please state whether you would be interested in attending another BJPsych Journal Club event in the future & if there are any other ways in which you feel the BJPsych could support you as a clinician or medical student *(free text)*
10) The information from this feedback form may be used anonymously when evaluating or writing reports regarding BJPsych journal clubs. Please indicate below whether you are happy for this information to be used *(free text)*
